# Garlic γ-glutamyl transpeptidases that catalyze deglutamylation of biosynthetic intermediate of alliin

**DOI:** 10.3389/fpls.2014.00758

**Published:** 2015-01-08

**Authors:** Naoko Yoshimoto, Ayami Yabe, Yuka Sugino, Soichiro Murakami, Niti Sai-ngam, Shin-ichiro Sumi, Tadamitsu Tsuneyoshi, Kazuki Saito

**Affiliations:** ^1^Graduate School of Pharmaceutical Sciences, Chiba UniversityChiba, Japan; ^2^Research Planning Department, Wakunaga Pharmaceutical CompanyAkitakata, Japan; ^3^Central Research Institute, Wakunaga Pharmaceutical CompanyAkitakata, Japan; ^4^RIKEN Center for Sustainable Resource ScienceYokohama, Japan

**Keywords:** γ-glutamyl transpeptidase, deglutamylation, alliin, secondary metabolism, garlic

## Abstract

*S*-Alk(en)yl-L-cysteine sulfoxides are pharmaceutically important secondary metabolites produced by plants that belong to the genus *Allium*. Biosynthesis of *S*-alk(en)yl-L-cysteine sulfoxides is initiated by *S*-alk(en)ylation of glutathione, which is followed by the removal of glycyl and γ-glutamyl groups and *S*-oxygenation. However, most of the enzymes involved in the biosynthesis of *S*-alk(en)yl-L-cysteine sulfoxides in *Allium* plants have not been identified. In this study, we identified three genes, *AsGGT1*, *AsGGT2*, and *AsGGT3*, from garlic (*Allium sativum*) that encode γ-glutamyl transpeptidases (GGTs) catalyzing the removal of the γ-glutamyl moiety from a putative biosynthetic intermediate of *S*-allyl-L-cysteine sulfoxide (alliin). The recombinant proteins of AsGGT1, AsGGT2, and AsGGT3 exhibited considerable deglutamylation activity toward a putative alliin biosynthetic intermediate, γ-glutamyl-*S*-allyl-L-cysteine, whereas these proteins showed very low deglutamylation activity toward another possible alliin biosynthetic intermediate, γ-glutamyl-*S*-allyl-L-cysteine sulfoxide. The deglutamylation activities of AsGGT1, AsGGT2, and AsGGT3 toward γ-glutamyl-*S*-allyl-L-cysteine were elevated in the presence of the dipeptide glycylglycine as a γ-glutamyl acceptor substrate, although these proteins can act as hydrolases in the absence of a proper acceptor substrate, except water. The apparent *K*_m_ values of AsGGT1, AsGGT2, and AsGGT3 for γ-glutamyl-*S*-allyl-L-cysteine were 86 μM, 1.1 mM, and 9.4 mM, respectively. Subcellular distribution of GFP-fusion proteins transiently expressed in onion cells suggested that AsGGT2 localizes in the vacuole, whereas AsGGT1 and AsGGT3 possess no apparent transit peptide for localization to intracellular organelles. The different kinetic properties and subcellular localizations of AsGGT1, AsGGT2, and AsGGT3 suggest that these three GGTs may contribute differently to the biosynthesis of alliin in garlic.

## INTRODUCTION

Production of cysteine-derived secondary metabolites, *S*-alk(en)yl-L-cysteine sulfoxides, is a pharmaceutically important characteristic of plants that belong to the genus *Allium*. These compounds are hydrolyzed by the endogenous vacuolar enzyme alliinase (EC. 4.4.1.4) upon tissue disruption to yield highly reactive alk(en)ylsulfenic acids that are spontaneously converted to various sulfur-containing compounds with diverse pharmacological activities, including antibacterial, antifungal, antivirus, immunostimulating, antioxidant, anticarcinogenic, antithrombotic, cholesterol- and triglyceride-lowering, and hypotensive effects (Jones et al., 2004; Rose et al., 2005; Iciek et al., 2009). To date, four major *S*-alk(en)yl-L-cysteine sulfoxides, *S*-allyl-L-cysteine sulfoxide (alliin), *S*-methyl-L-cysteine sulfoxide (methiin), *S*-*trans*-1-propenyl-L-cysteine sulfoxide (isoalliin), and *S*-propyl-L-cysteine sulfoxide (propiin), have been identified and isolated from *Allium* plants (Jones et al., 2004; Rose et al., 2005).

**FIGURE 1:**
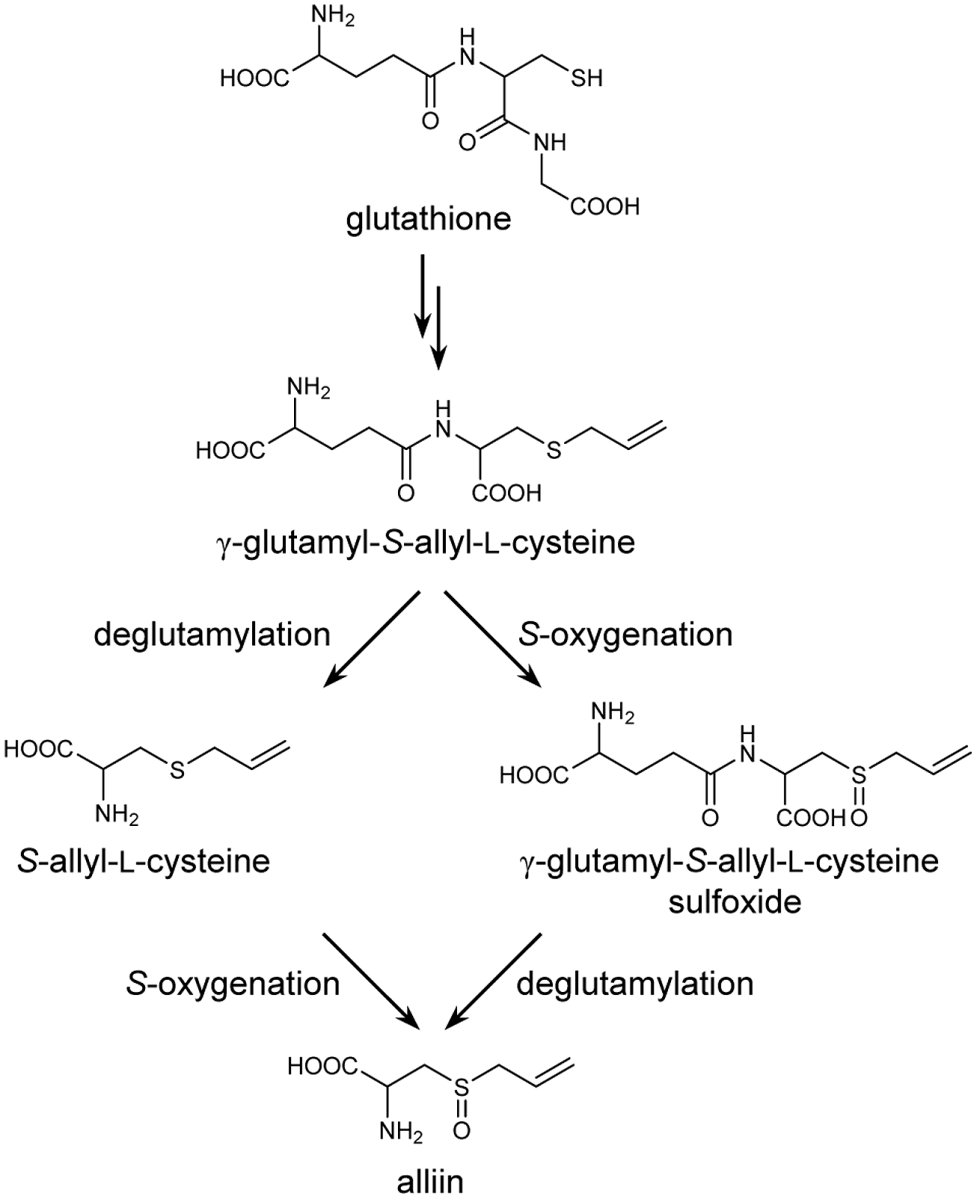
**Proposed biosynthetic pathway for alliin in garlic**.

Biosynthesis of *S*-alk(en)yl-L-cysteine sulfoxides in *Allium* plants has previously been proposed to proceed via glutathione *S*-conjugates, according to the results of precursor feeding and pulse radiolabeling experiments (Suzuki et al., 1962; Turnbull et al., 1980; Lancaster and Shaw, 1989). In the proposed pathway, glutathione is *S*-alk(en)ylated at the cysteine residue, followed by the removal of a glycyl group to form a biosynthetic intermediate, γ-glutamyl-*S*-alk(en)yl-L-cysteine. This γ-glutamylated sulfide compound is further deglutamylated and *S*-oxygenated to yield *S*-alk(en)yl-L-cysteine sulfoxide (**Figure 1**). Although the results of pulse radiolabeling suggest that *S*-oxygenation may likely occur before deglutamylation in onion (*Allium cepa*; Lancaster and Shaw, 1989), the order of *S*-oxygenation and deglutamylation in other *Allium* plants remains unclear.

γ-Glutamyl transpeptidase (GGT; EC 2.3.2.2), also known as γ-glutamyl transferase, is the enzyme that catalyzes the transfer of the γ-glutamyl moiety of γ-glutamyl compounds to amino acids, short peptides (transpeptidation), or water (hydrolysis; Tate and Meister, 1981). The physiological role of GGT, commonly observed in bacteria, yeast, animals, and plants, is the catabolism of glutathione. *Saccharomyces cerevisiae* has a vacuolar GGT protein responsible for the degradation of glutathione in the vacuole during nitrogen starvation in order to supply the constituent amino acids of glutathione to the starved cell (Mehdi and Penninckx, 1997), whereas GGTs in *Escherichia coli*, mammals, and plants function in the breakdown of extracellular glutathione (Suzuki et al., 1999; Storozhenko et al., 2002; Dominici et al., 2005; Martin et al., 2007; Ohkama-Ohtsu et al., 2007a). GGT is also responsible for degrading glutathione-related compounds. For example, GGT is involved in the biosynthesis of the phytoalexin camalexin by removing a γ-glutamyl group from glutathione-indole-3-acetonitrile in *Arabidopsis* (Su et al., 2011), in the conversion of the endogenous glutathione *S*-conjugate leukotriene C4 to leukotriene D4 in rats (Anderson et al., 1982), and in the glutathione-mediated detoxification of xenobiotics in both animals and plants (Zhang et al., 2005; Grzam et al., 2007; Ohkama-Ohtsu et al., 2007b). Given that the removal of a γ-glutamyl group from the biosynthetic intermediate γ-glutamyl-*S*-alk(en)yl-L-cysteine is required for the biosynthesis of *S*-alk(en)yl-L-cysteine sulfoxides in the genus *Allium*, the involvement of GGTs in the biosynthesis of *S*-alk(en)yl-L-cysteine sulfoxides as deglutamylation enzymes has been proposed. The fact that the levels of biosynthetic intermediate γ-glutamyl peptides were decreased while GGT activity was increased during sprouting in onion bulbs also supports this idea (Lancaster and Shaw, 1991). To date, several efforts have been made to identify and characterize GGTs in *Allium* plants. A GGT partially purified from onion showed high substrate specificity toward γ-glutamyl compounds that are putative intermediates of *S*-alk(en)yl-L-cysteine sulfoxide biosynthesis, strongly suggesting the involvement of this GGT in the biosynthesis of *S*-alk(en)yl-L-cysteine sulfoxides (Lancaster and Shaw, 1994). Recently, a GGT protein was purified to homogeneity from sprouting onion bulbs, and a partial cDNA for this GGT, *AcGGT*, was cloned; however, in contrast to the previously partially purified onion GGT (Lancaster and Shaw, 1994), the purified AcGGT protein showed high affinity for glutathione and glutathione *S*-conjugates but could not utilize γ-glutamyl-*trans*-*S*-1-propenyl-L-cysteine sulfoxide as a good γ-glutamyl donor substrate, suggesting that AcGGT is not the major enzyme catalyzing deglutamylation in the biosynthesis of *S*-alk(en)yl-L-cysteine sulfoxides in onion (Shaw et al., 2005). A partial cDNA of *AsGGT*, which has high sequence homology to *AcGGT*, was isolated from garlic (*Allium sativum*), and its mRNA expression patterns suggested that AsGGT may play a role in synthesizing *S*-alk(en)yl-L-cysteine sulfoxides in garlic cloves during cold storage (Cho et al., 2012).

In this study, we cloned three genes encoding GGTs, *AsGGT1*, *AsGGT2*, and *AsGGT3*, that are suggested to be involved in the biosynthesis of alliin in garlic. The substrate preferences of AsGGT1, AsGGT2, and AsGGT3 suggest that a key biosynthetic intermediate, γ-glutamyl-*S*-allyl-L-cysteine, is deglutamylated by these GGTs prior to being *S*-oxygenated during alliin biosynthesis in garlic.

## MATERIALS AND METHODS

### PLANT MATERIALS AND REAGENTS

Total RNA was extracted from the bulbs of *A. sativum* L. ‘Fukuchi-howaito’. *S*-Allyl-L-cysteine was purchased from Tokyo Chemical Industry (Tokyo, Japan). Alliin [(*R*_C_*S*_S_)-*S*-allyl-L-cysteine sulfoxide] was synthesized and purified according to previously reported methods (Yu et al., 1994; Kubec et al., 1999; Kubec and Dadáková, 2008). γ-Glutamyl-*S*-allyl-L-cysteine was synthesized as follows. A mixture of 2.59 g of *N*-phthaloyl-L-glutamic anhydride and 1.93 g of *S*-allyl-L-cysteine in 10 mL of acetic acid was stirred at 60°C for 2 h. The solvent in the reaction mixture was removed in vacuo, and the residue was suspended in ethyl acetate and washed with brine. The organic layer was dried using sodium sulfate, and the solvent was removed in vacuo. After 1.5 g of residue was dissolved in 10 mL of methanol, 0.15 mL of hydrazine monohydrate was added, and this mixture was refluxed at 80°C for 1 h. Solvent was removed in vacuo, and the residue was washed with ethanol. The residue was recrystallized in a mixture of ethanol and water. The crystalline powder was applied to Dowex^TM^ 50Wx8 (The Dow Chemical Company, USA), and the column eluate and rinsing were combined and lyophilized. γ-Glutamyl-*S*-allyl-L-cysteine sulfoxide was synthesized as follows. γ-Glutamyl-*S*-allyl-L-cysteine was dissolved in water, and 1.1 equimolar of hydrogen peroxide was added. The mixture was stirred at room temperature, and the solvent was removed in vacuo. The residue was dried under reduced pressure with phosphorus (V) oxide at room temperature. Structures of synthesized γ-glutamyl-*S*-allyl-L-cysteine and γ-glutamyl-*S*-allyl-L-cysteine sulfoxide were confirmed using ^13^C-NMR and ^1^H-NMR. All other chemicals were of analytical grade and were purchased from Sigma (St. Louis, MO, USA), Nacalai Tesque (Kyoto, Japan), or Wako Pure Chemical Industries (Osaka, Japan).

### CLONING OF *AsGGT1*, *AsGGT2*, AND *AsGGT3* FROM GARLIC

Molecular biological experiments were performed according to the standard protocols (Sambrook et al., 1989), unless otherwise specified. Total RNA was extracted from garlic cloves by using the RNeasy plant mini kit (Qiagen, Valencia, CA, USA) and treated with DNase I (Life Technologies, Carlsbad, CA, USA). Reverse transcription (RT) was performed using SuperScript II reverse transcriptase (Life Technologies) and oligo-d(T)_12-18_. Partial cDNAs of *AsGGT1* and *AsGGT2* were amplified by PCR using *Ex*Taq DNA polymerase (Takara, Tokyo, Japan) and oligonucleotide primers designed from the nucleotide sequences of two garlic EST clones, i.e., EPP005LLAA12S004013 and EPP005LLAA12S003688 in GarlicESTdb (Kim et al., 2009^1^): AsGGT1-Core-F (5′-ATCGCCACTTCATATGAACC-3′) and AsGGT1-Core-R (5′-GATAATGCTAGATATGGCTC-3′) for *AsGGT1*; AsGGT2-Core-F (5′-CTCCTCCACATTAATGGAAC-3′) and AsGGT2-Core-R (5′-AAGTGGTCCCAAACATTTGTC-3′) for *AsGGT2*. For the amplification of a partial region of *AsGGT3* cDNA, degenerate primers designed based on the sequences of conserved regions of known GGTs, GGT-degenerate-F (5′-ATHGTNYTNAAYAAYGARATG-3′) and GGT-degenerate-R (5′-CCNCCYTTNCKNGGRTC-3′), were used. Rapid amplification of cDNA ends (RACE) was performed using 5′-Full RACE Core Set (Takara) and 3′-Full RACE Core Set (TaKaRa), according to the manufacturer’s protocols. 5′-RACE was performed using the following primers: AsGGT1-5′-RACE-RT (5′-[Phos]TCTTCTGAACCG-3′), AsGGT1-5′-RACE-F1(5′-TGCTCTCACCACTCTGTTC-3′), AsGGT1-5′-RACE-F2 (5′-GACTCCATCTCTCATCAGTTC-3′), As GGT1-5′-RACE-R1 (5′-TCACGAACGATGAGCGATG-3′), and AsGGT1-5′-RACE-R2 (5′-CCAGTTTCTGATCAGAAGAAGC-3′) for *AsGGT1*; AsGGT2-5′-RACE-RT (5′-[Phos]TGAGCTCGTAA ACTC-3′), AsGGT2-5′-RACE-F1 (5′-TGTGCGACGGTATCCG ATCA-3′), AsGGT2-5′-RACE-F2 (5′-CTCAATCCAATTCAACCT AGAC-3′), AsGGT2-5′-RACE-R1 (5′-CATTGTGCAGCGGACGA TAG-3′), and AsGGT2-5′-RACE-R2 (5′-GGTTCCATTAATGTGG AGGAG-3′) for *AsGGT2*; AsGGT3-5′-RACE-RT (5′-[Phos]GTATC CATCGGGAAT-3′), AsGGT3-5′-RACE-F1 (5′-TGAAAAAGAAA GGGCAGCTC-3′), AsGGT3-5′-RACE-F2 (5′-GGTTTAGGGATT GCAAATGG-3′), AsGGT3-5′-RACE-R1 (5′-CCTCCACTTGCGC CTAGAG-3′), and AsGGT3-5′-RACE-R2 (5′-GGTGGCGGCATAT TGTTATT-3′) for *AsGGT3*. 3′-RACE was performed using 3 sites adaptor primer (5′-CTGATCTAGAGGTACCGGATCC-3′) and the following gene-specific primers: AsGGT1-3′-RACE-F1 (5′-AGCTGGTCTACATGCTGCATGG-3′) and AsGGT1-3′-RACE-F2 (5′-TCCCATGGAAGTCACTTTTCG-3′) for *AsGGT1*; AsGGT2-3′-RACE-F1 (5′-GCTTTTGATGCTAGAGAGACTGC-3′) and AsGGT2-3′-RACE-F2 (5′-ATCACTCCGACAAATGTTTG-3′) for *AsGGT2*; AsGGT3-3′-RACE-F (5′-TGAAAAAGAAAGGGCAGC TC-3′) for *AsGGT3*. cDNA clones of *AsGGT1*, *AsGGT2*, and *AsGGT3* were re-isolated by RT-PCR using KOD plus DNA polymerase (Toyobo, Osaka, Japan) and the following primers: AsGGT1-F (5′-TCATATTCTGACGCAGATTCCACAG-3′) and AsGGT1-R (5′-TGTTCAATCATATTTTGTACAAATAGAC-3′) for *AsGGT1*; AsGGT2-F (5′-CGAGCAAATTAATTCATTTTGGCTC AC-3′) and AsGGT2-R (5′-GCATACCAATCGCCACAAACTC-3′) for *AsGGT2*; AsGGT3-F (5′-GTTAACAACAGGATTGGTCAATG CTC-3′) and AsGGT3-R (5′-CAGCAAACAACGCACTATTCAGT TTCTG-3′) for *AsGGT3*.

### HETEROLOGOUS EXPRESSION OF AsGGT1, AsGGT2, AND AsGGT3 IN YEAST

The coding regions of *AsGGT1*, *AsGGT2*, and *AsGGT3* were amplified by PCR using the cloned cDNA fragments described above, KOD plus DNA polymerase (Toyobo), and the following gene-specific primers: AsGGT1-FKpn3A (5′-GGTACCAAAATGAACCAAATGGCGCCGGCTTC-3′) and AsGGT1-stop-RXh (5′-CTCGAGCTATACACAAGCAGGACTTC CATC-3′) for *AsGGT1*; AsGGT2-FKpn3A (5′-GGTACCAAAATG GAACCGGCGCATGATGACTTAG-3′) and AsGGT2-stop-RXh (5′-CTCGAGTCACACACATGCAGGACTTCCATC-3′) for *AsGG T2*; AsGGT3-FKpn3A (5′-GGTACCAAAATGCTAATTAATTCATA CCCTGC-3′) and AsGGT3-stop-RXh (5′-CTCGAGTCAGTATCC ATCGGGAATACC-3′) for *AsGGT3*. The underlined sequences in the primers correspond to *Kpn*I and *Xho*I restriction sites for subcloning. The amplified fragments were cloned into the pGEM-T easy vector (Promega, Madison, WI, USA). After their nucleotide sequences were confirmed, the coding regions of *AsGGT1*, *AsGGT2*, and *AsGGT3* were cut out as *Kpn*I-*Xho*I fragments and were inserted between the *Kpn*I and *Xho*I sites in the yeast expression vector pYES2 (Life Technologies). The resulting plasmids, pYES2-AsGGT1, pYES2-AsGGT2, and pYES2-AsGGT3, and pYES2 empty vector were transformed into the *Saccharomyces cerevisiae* mutant strain BJ2168 (*MATa, prb1-1122, prc1-407, pep4-3, ura3-52, leu2, trp1*; Nippon Gene, Tokyo, Japan) by using the lithium acetate method (Gietz et al., 1992). The transformants were selected on SD minimal medium (Sherman, 1991) containing no uracil. For the induction of recombinant proteins, the yeast cells grown in SD minimal medium without uracil at 28°C for 1 days were transferred to 10 volumes of uracil-less SD medium containing 2% (w/v) galactose instead of glucose to activate the *GAL1* promoter on pYES2, and cultured at 28°C for 1 days. The cells were harvested and disrupted at 4°C with 425–600-μm (diameter) glass beads in buffer G [10 mM Tris-HCl (pH 7.5), 300 mM sorbitol, 100 mM NaCl, 5 mM MgCl_2_, 1 mM EDTA, and 1 μM pepstatin A]. The lysate was centrifuged at 10,000 × *g* for 5 min, and the supernatant was collected. Buffer G of the supernatant was subsequently replaced with 50 mM Tris-HCl (pH 8.0) by using the Sephadex column PD Mini Trap G-25 (GE Healthcare, Uppsala, Sweden), according to the manufacturer’s protocol. The eluted yeast crude proteins were used for the enzymatic activity assay described below. Protein concentrations were determined using the Bio-Rad protein assay (Bio-Rad, CA, USA) based on the Bradford method (Bradford, 1976), using bovine serum albumin as the standard.

### ASSAYS OF GGT ENZYME ACTIVITIES

Assays of GGT enzyme activities were performed by analyzing the amount of deglutamylated compounds produced from γ-glutamylated compounds by yeast crude proteins in 6 h at 37°C. The amount of deglutamylated compounds increased linearly over the 6-h incubation period.

Deglutamylation activities using γ-glutamyl-*p*-nitroanilide as the substrate were determined spectrophotometrically according to a previously described method (Orlowski and Meister, 1963), with slight modifications, as follows: the reaction mixture, which consisted of 0.0125 μg μl^-1^ yeast crude protein, 50 mM Tris-HCl (pH 8.0), 10 mM glycylglycine, and 1 mM γ-glutamyl-*p*-nitroanilide, was incubated for 6 h at 37°C, and *p*-nitroaniline released from γ-glutamyl-*p*-nitroanilide was monitored at 412 nm.

For the analysis of deglutamylation activities toward γ-glutamyl-*S*-allyl-L-cysteine and γ-glutamyl-*S*-allyl-L-cysteine sulfoxide, the enzyme assay reaction mixture consisted of 0.67 μg μl^-1^ yeast crude protein, 50 mM Tris-HCl (pH 8.0), 10 mM glycylglycine, and 1 mM γ-glutamyl-*S*-allyl-L-cysteine or γ-glutamyl-*S*-allyl-L-cysteine sulfoxide was incubated for 6 h at 37°C. For the determination of the enzyme activity in a pH range of 6.0–7.0, the reaction mixture containing 50 mM 2-(*N*-morpholino)ethanesulfonic acid buffer, instead of 50 mM Tris-HCl buffer, was used. For the determination of the enzyme activity in a pH range of 7.0–9.0, 50 mM Tris-HCl buffer at pH values from 7.0 to 9.0, instead of 50 mM Tris-HCl (pH 8.0), was used. For the analysis of the effects of glycylglycine as a γ-glutamyl acceptor, deglutamylation activity was determined in the reaction mixture with or without 10 mM glycylglycine. The reaction was initiated by the addition of yeast crude proteins. After incubation at 37°C, proteins in the reaction mixture were removed using a centrifugal ultrafiltration device (molecular weight cut-off, 10 kD; Kurabo, Osaka, Japan). *S*-Allyl-L-cysteine and alliin in the ultrafiltrated solution were quantified using high-performance liquid chromatography (HPLC). For the kinetic analysis, assays were carried out with γ-glutamyl-*S*-allyl-L-cysteine concentrations ranging from 12.5 to 1000 μM for AsGGT1, 0.5–8 mM for AsGGT2, and 1–25 mM for AsGGT3. *K*_m_ values were calculated from triplicate date sets according to the Michaelis-Menten equation.

### ANALYSIS OF SULFUR-CONTAINING METABOLITES BY USING HPLC

The enzymatic products were analyzed quantitatively by using HPLC (Hitachi, Tokyo, Japan) with the cation-exchange column (TSKgel Aminopak, Tosoh, Tokyo, Japan). For the determination of *S*-allyl-L-cysteine, a mobile phase composed of 67 mM sodium citrate, 8% (v/v) ethanol, and 0.01% (v/v) octanoic acid (pH 3.26) was used for an isocratic elution. The column temperature was 35°C. For the determination of alliin, the mobile phase consisted of 22 mM trisodium citrate and 80 mM citric acid and the column temperature was 40°C. After separation, *S*-allyl-L-cysteine and alliin were fluorescently derivatized using *o*-phthalaldehyde and were detected using a fluorescence detector (excitation 340 nm, emission 455 nm). Identification of *S*-allyl-L-cysteine and alliin in the enzymatic reaction mixture was based on comparisons of retention times of the synthesized standards.

### SUBCELLULAR LOCALIZATION ANALYSIS

For the construction of the fusion gene constructs of *35S_pro_:As GGT1_N100_:GFP*, *35S_pro_:AsGGT2_N100_:GFP*, and *35S_pro_:AsGGT3_N100_ :GFP*, partial coding regions of *AsGGT1*, *AsGGT2*, and *AsGGT3* that encode the N-terminal 100 amino acid residues were amplified by PCR using KOD plus DNA polymerase (Toyobo) and the following gene-specific primers: AsGGT1-FSal (5′-GTCGACATGAACCAAATGGCGCCGGCTTCTTC-3′) and AsGGT1-N100-RNco (5′-CCATGGAACCACCACCACCACC ACCTTTTCTCAGAACTGAAGCTCC-3′) for *AsGGT1*; AsGGT2-FSal (5′-GTCGACATGGAACCGGCGCATGATGACTTAG-3′) and AsGGT2-N100-RNco (5′-CCATGGAACCACCACCACCACCACC TAAAGCGTCCACAGCATGACC-3′) for *AsGGT2*; AsGGT3-FSal (5′-GTCGACATGCTAATTAATTCATACCCTGCATATC-3′) and AsGGT3-N100-RNco (5′-CCATGGAACCACCACCACCACCACC ACTAACAACTCCTAAACAAAATG-3′) for *AsGGT3*. The sequence encoding hexa-Gly residues was generated downstream of the sequence of *AsGGT1*, *AsGGT2*, and *AsGGT3* by the PCR. The underlined sequences in the primers correspond to *Sal*I and *Nco*I restriction sites for subcloning. The amplified DNA fragments were cloned into the pGEM-T easy vector (Promega) to confirm the nucleotide sequence. Partial *AsGGT1*, *AsGGT2*, and *AsGGT3*, fused with the sequence encoding hexa-Gly residues, were cut out as *Sal*I-*Nco*I fragments and were inserted between the *Sal*I and *Nco*I sites in pTH2 (Chiu et al., 1996). Each of the resulting plasmids was co-introduced with pDsRed plasmid (Kitajima et al., 2009) into onion epidermal cells by particle bombardment at 150 psi, using a Helios gene gun (Bio-Rad). After bombardment, onion peels were incubated for 26–47 h on B5 medium (Gamborg et al., 1968) in the dark at 25°C. GFP and DsRed fluorescence in the onion cells were observed using a LSM710 confocal laser-scanning microscope (Carl Zeiss, Oberkochen, Germany).

### PHYLOGENETIC ANALYSIS

Phylogenetic analysis was performed using MEGA version 6 software (Tamura et al., 2013) based on the ClustalW multiple alignment. A phylogenetic tree was generated using the neighbor-joining method.

## RESULTS

### IDENTIFICATION OF THREE GENES ENCODING γ-GLUTAMYL TRANSPEPTIDASES IN GARLIC

We found two garlic EST clones, EPP005LLAA12S004013 and EPP005LLAA12S003688, that show high sequence homology with known GGTs in GarlicESTdb (Kim et al., 2009 ^2^). Utilizing the sequence information on these EST clones, we obtained two different full-length cDNA clones by 5′- and 3′-RACE and RT-PCR from the RNA of garlic cloves and designated them as *AsGGT1* (GenBank Accession No. LC008010) and *AsGGT2* (GenBank Accession No. LC008011). In addition, we amplified one garlic cDNA fragment using degenerate primers designed based on the conserved regions of known plant GGTs. A full-length cDNA clone was obtained by RACE and RT-PCR, and was designated as *AsGGT3* (GenBank Accession No. LC008012). The cDNAs of *AsGGT1*, *AsGGT2*, and *AsGGT3* coded for polypeptides of 627, 622, and 605 amino acids, respectively. The deduced amino acid sequences of *AsGGT1* and *AsGGT2* shared 69% identity, whereas the amino acid sequence identity of *AsGGT3* with *AsGGT1* and *AsGGT2* was 46 and 43%, respectively. The amino acid sequence of *AsGGT3* showed 99% sequence identity with that of a partial sequence of garlic *AsGGT* (Cho et al., 2012) in their 158 aa overlapped region and showed 92% sequence identity with that of a partial sequence of onion *AcGGT* (Shaw et al., 2005) in their 543 aa overlapped region. *Arabidopsis thaliana* has three functional GGTs, AtGGT1, AtGGT2, and AtGGT4. Among them, AtGGT4 is known to have a long N-terminal sequence that determines vacuolar localization, compared to AtGGT1 and AtGGT2 that localize in the extracellular space (Grzam et al., 2007; Ohkama-Ohtsu et al., 2007b). As in *Arabidopsis* AtGGT4, the deduced amino acid sequences of *AsGGT1*, *AsGGT2*, and *AsGGT3* had longer N-terminal sequences than those of *Arabidopsis* AtGGT1 and AtGGT2, suggesting the presence of the N-terminal signal sequences for targeting to cellular organelles in AsGGT1, AsGGT2, and AsGGT3. N-terminal regions of the deduced amino acid sequences of *AsGGT1*, *AsGGT2*, and *AsGGT3* were not highly similar to each other or to that of AtGGT4, despite the high sequence similarities among the rest of their regions.

**FIGURE 2:**
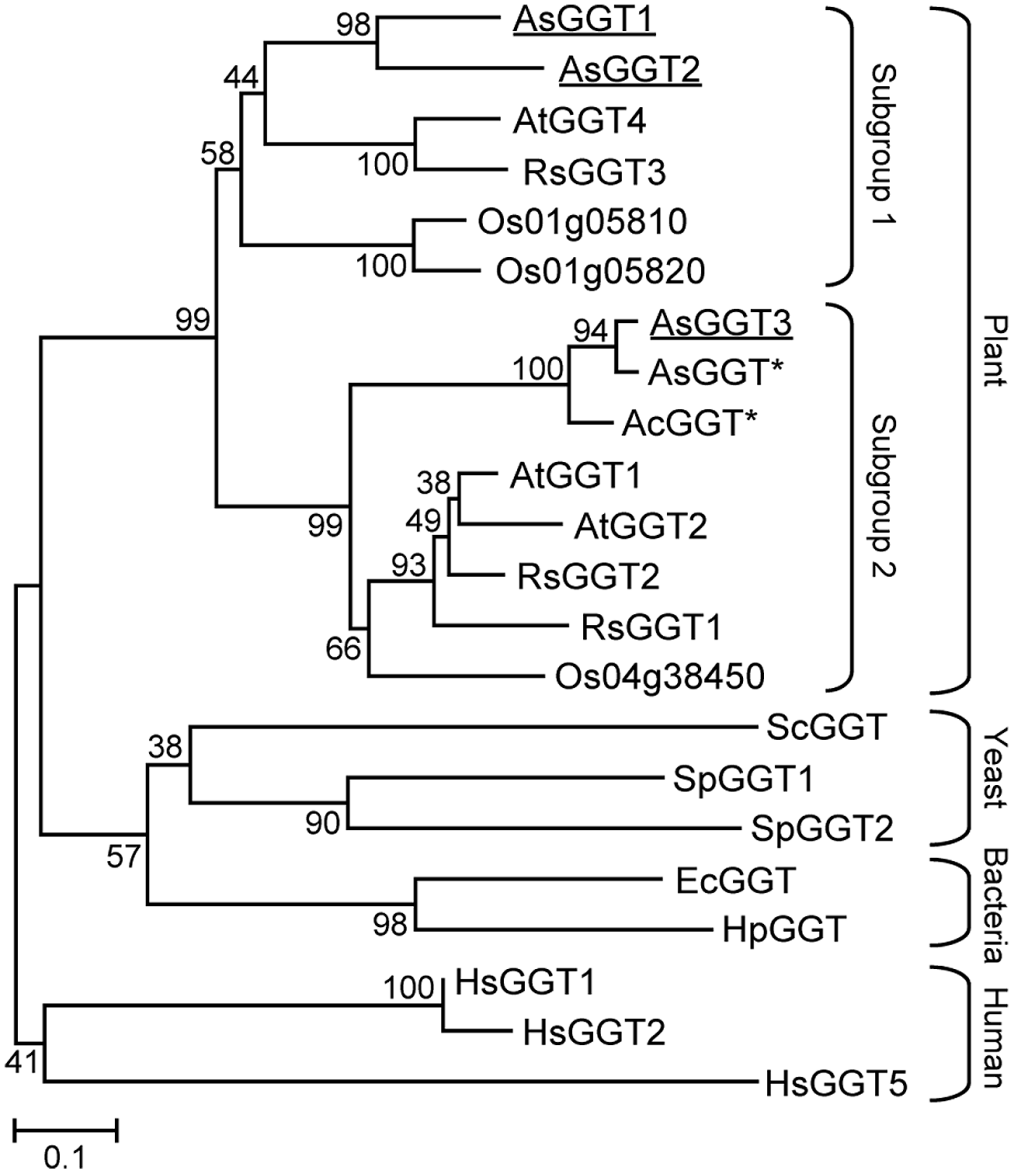
**Phylogenetic tree for the γ-glutamyl transpeptidases.** An unrooted tree was constructed using MEGA version 6 software based on the ClustalW multiple alignment. Bootstrap values (1000 replicates) are shown next to the branches. Plant GGTs are classified into two subgroups. Garlic AsGGT1, AsGGT2, and AsGGT3 analyzed in this study are underlined. Asterisks indicate partial amino acid sequences. Abbreviations for species are: Ac, *Allium cepa*; As, *Allium sativum*; At, *Arabidopsis thaliana*; Ec, *Escherichia coli*; Hp, *Helicobacter pylori*; Hs, *Homo sapiens*; Os, *Oryza sativa*; Rs, *Raphanus sativus*; Sc, *Saccharomyces cerevisiae*; Sp, *Schizosaccharomyces pombe*. The GenBank accession numbers for the sequences are shown in parentheses: AcGGT (AAL61611); AsGGT1 (LC008010); AsGGT2 (LC008011); AsGGT3 (LC008012); AtGGT1 (AEE87097); AtGGT2 (AEE87099); AtGGT4 (AEE85602); EcGGT (AAA23869); HpGGT (AAD08162); HsGGT1 (AAH25927); HsGGT2 (XP_006724458); HsGGT5 (AAH73999); Os01g05810 (BAD61112); Os01g05820 (BAD61113); Os04g38450 (CAD40892); RsGGT1 (BAC45233); RsGGT2 (BAC56855); RsGGT3 (BAD22536); ScGGT (DAA09609); SpGGT1 (AAN01227); SpGGT2 (AAQ57121).

A phylogenic tree was generated by the neighbor-joining method, using the amino acid sequences of known GGTs from plants, yeast, bacteria, and humans (**Figure 2**). All plant GGTs were classified into the same branch, which was further divided into two distinct subgroups. AsGGT1 and AsGGT2 belonged to the subgroup containing *Arabidopsis* AtGGT4 that functions in the degradation of glutathione *S*-conjugates in the vacuole (Grzam et al., 2007; Ohkama-Ohtsu et al., 2007b), whereas AsGGT3 belonged to the subgroup containing *Arabidopsis* AtGGT1 and AtGGT2 that function in the breakdown of extracellular glutathione (Martin et al., 2007; Ohkama-Ohtsu et al., 2007a) together with onion AcGGT (Shaw et al., 2005).

Generally, in bacteria, yeast, plants, and mammals, GGT is a heteromeric protein consisting of large and small subunits, both of which are generated from a common inactive precursor polypeptide by autoprocessing (Penninckx and Jaspers, 1985; Storozhenko et al., 2002; Suzuki and Kumagai, 2002; Ikeda and Taniguchi, 2005; Shaw et al., 2005; Nakano et al., 2006; Boanca et al., 2007). Some plants, such as tomato, onion, and radish, are suggested to have GGT proteins consisting of a single polypeptide, although their sequence information remains unknown (Lancaster and Shaw, 1994; Martin and Slovin, 2000; Nakano et al., 2006). The deduced amino acid sequences of *AsGGT1*, *AsGGT2*, and *AsGGT3* possessed the conserved threonine residue required for autocatalytic processing and the amino acid residues necessary for GGT activity, which were previously identified by biochemical and structural analyses of GGTs from humans and *E. coli* (Ikeda et al., 1993, 1995a,b; Okada et al., 2006).

### *IN VITRO* CHARACTERIZATION OF RECOMBINANT AsGGT1, AsGGT2, AND AsGGT3

Recombinant proteins of AsGGT1, AsGGT2, and AsGGT3 were independently expressed in budding yeast, and the crude protein extracts were used for the *in vitro* enzymatic activity assays. To confirm whether recombinant proteins of AsGGT1, AsGGT2, and AsGGT3 were expressed as mature GGT enzymes in yeast cells, we first examined deglutamylation activities of these recombinant proteins by using the standard procedure that utilizes γ-glutamyl-*p*-nitroanilide, a common synthetic γ-glutamyl donor substrate for known GGTs (Orlowski and Meister, 1963). For a γ-glutamyl acceptor substrate, we used dipeptide glycylglycine. Crude protein extracts from control yeast carrying the empty vector converted γ-glutamyl-*p*-nitroanilide to *p*-nitroaniline (**Table 1**), showing that yeast endogenous GGT could utilize γ-glutamyl-*p*-nitroanilide as a γ-glutamyl donor substrate, as reported previously (Payne and Payne, 1984). The amounts of *p*-nitroaniline released from γ-glutamyl-*p*-nitroanilide in assays using crude protein extracts from yeast expressing AsGGT1, AsGGT2, and AsGGT3, respectively, were significantly higher than that in assays using crude protein extracts from control yeast (**Table 1**), indicating that the recombinant proteins of AsGGT1, AsGGT2, and AsGGT3 were successfully expressed and folded to form mature functional GGT proteins that can utilize γ-glutamyl-*p*-nitroanilide as a γ-glutamyl donor substrate in yeast cells.

**Table 1:** Specificity of AsGGT1, AsGGT2, and AsGGT3 for γ-glutamyl donor substrates.

Substrate	GGT activity (pmol μg^-1^ protein hr^-1^)			
	Empty vector	AsGGT1	AsGGT2	AsGGT3
γ-glutamyl-*p*-nitroanilide	52.0 ± 3.6	135.9 ± 10.7	103.6 ± 2.2	130.4 ± 6.0
γ-glutamyl-*S*-allyl-L-cysteine	ND	30.5 ± 0.2	17.4 ± 1.2	35.6 ± 2.5
γ-glutamyl-*S*-allyl-L-cysteine sulfoxide	ND	1.6 ± 0.0	ND	1.0 ± 0.0

*Each γ-glutamyl donor substrate was used at a concentration of 1 mM. Activities were measured in the presence of glycylglycine as the γ-glutamyl acceptor substrate. Data represent the mean ± SD (*n* = 4 for γ-glutamyl-*p*-nitroanilide, *n* = 3 for γ-glutamyl-*S*-allyl-L-cysteine, and *n* = 3 for γ-glutamyl-*S*-allyl-L-cysteine sulfoxide). ND means no detectable activity*.

**FIGURE 3:**
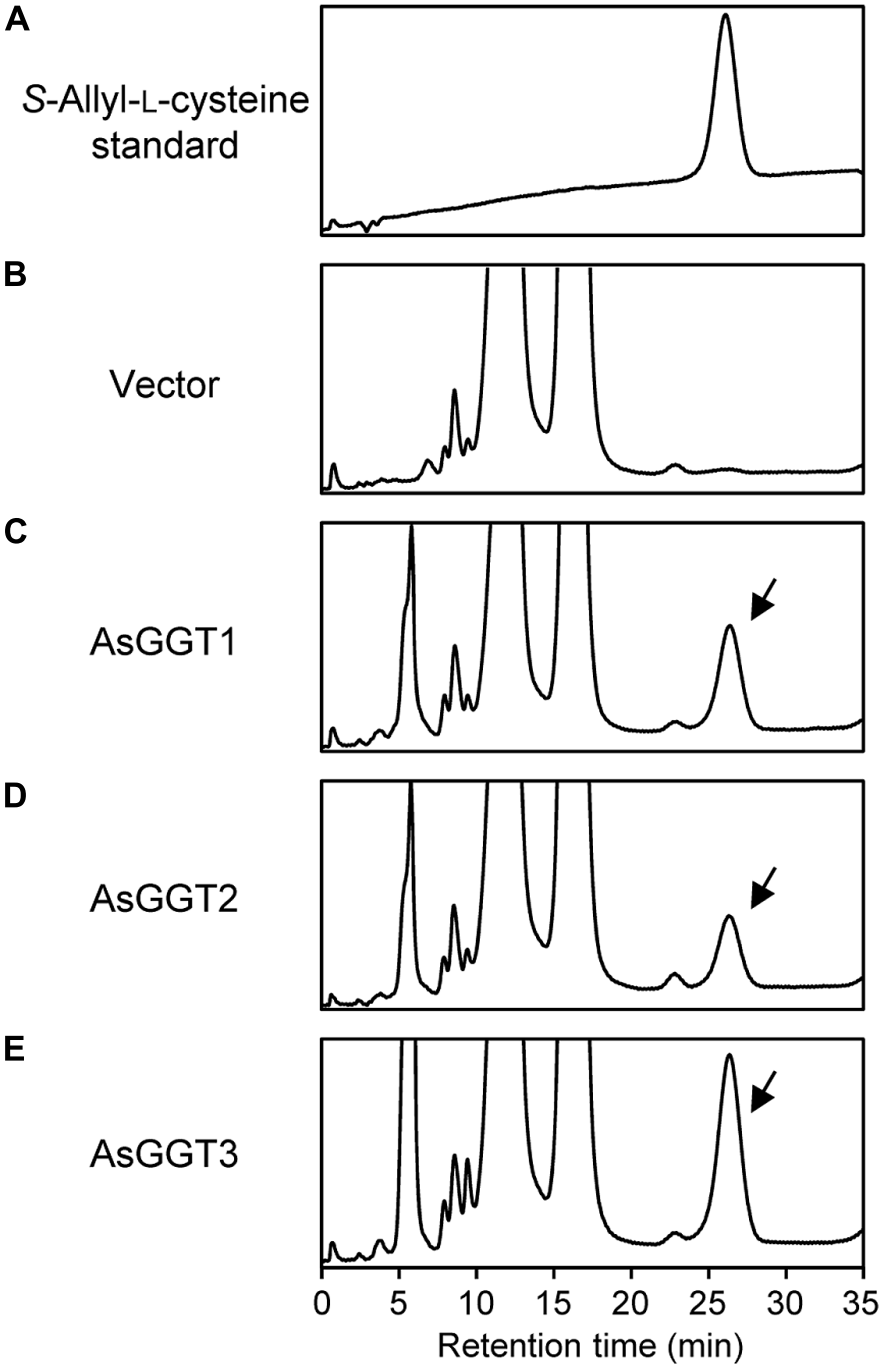
**Deglutamylation activities of recombinant AsGGT1, AsGGT2, and AsGGT3 toward γ-glutamyl-*S*-allyl-L-cysteine.** High-performance liquid chromatography (HPLC) elution profiles of the *S*-allyl-L-cysteine standard **(A)** and the reaction products from γ-glutamyl-*S*-allyl-L-cysteine by the crude protein extracts of yeast carrying empty vector **(B)** or yeast expressing AsGGT1 **(C)**, AsGGT2 **(D)**, and AsGGT3 **(E)** are shown. Arrows indicate peaks of *S*-allyl-L-cysteine in the reaction products.

**FIGURE 4:**
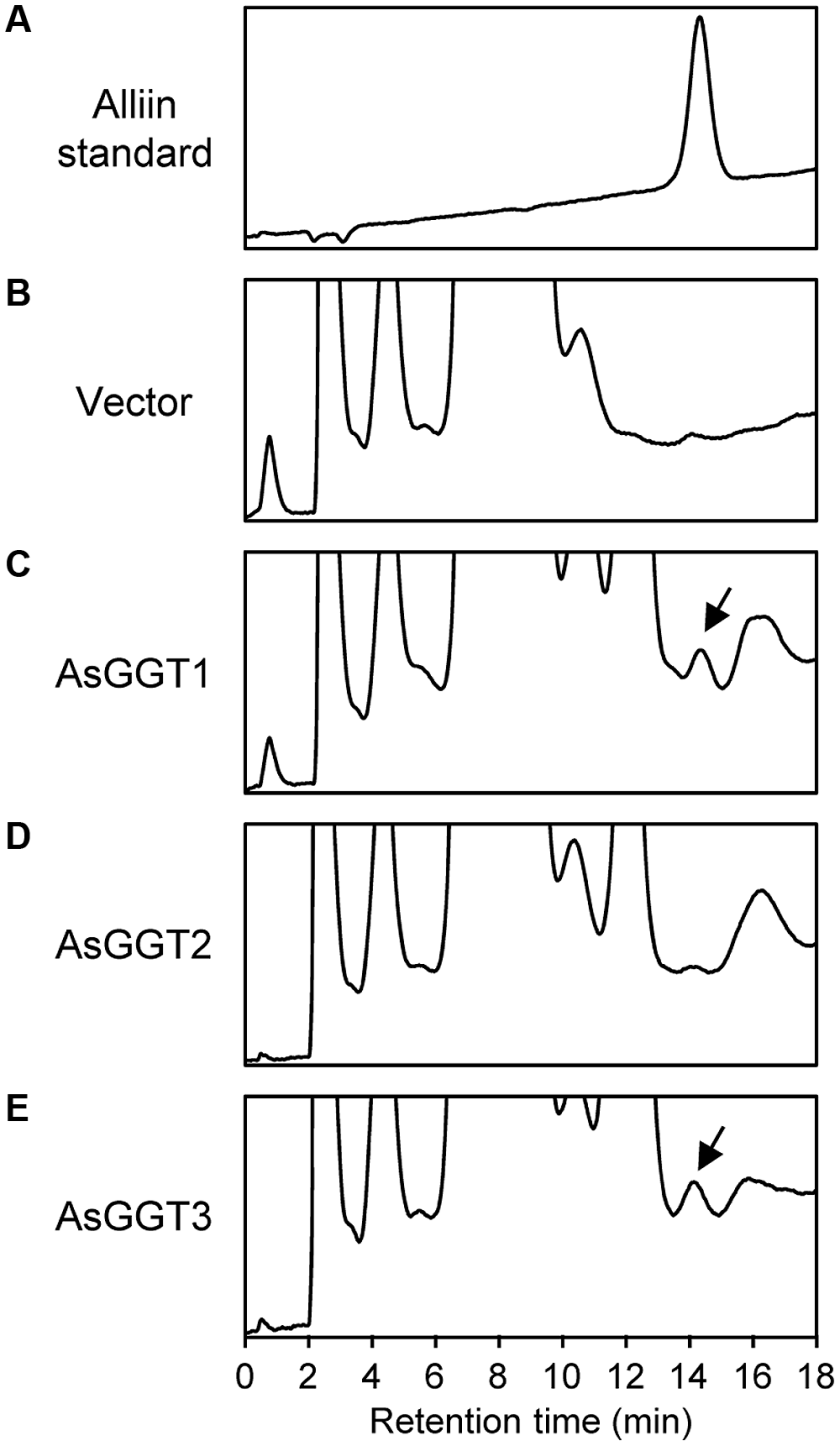
**Deglutamylation activities of recombinant AsGGT1, AsGGT2, and AsGGT3 toward γ-glutamyl-*S*-allyl-L-cysteine sulfoxide.** HPLC elution profiles of the alliin standard **(A)** and the reaction products from γ-glutamyl-*S*-allyl-L-cysteine sulfoxide by the crude protein extracts of yeast carrying empty vector **(B)** or yeast expressing AsGGT1 **(C)**, AsGGT2 **(D)**, and AsGGT3 **(E)** are shown. Arrows indicate peaks of alliin in the reaction products.

**FIGURE 5:**
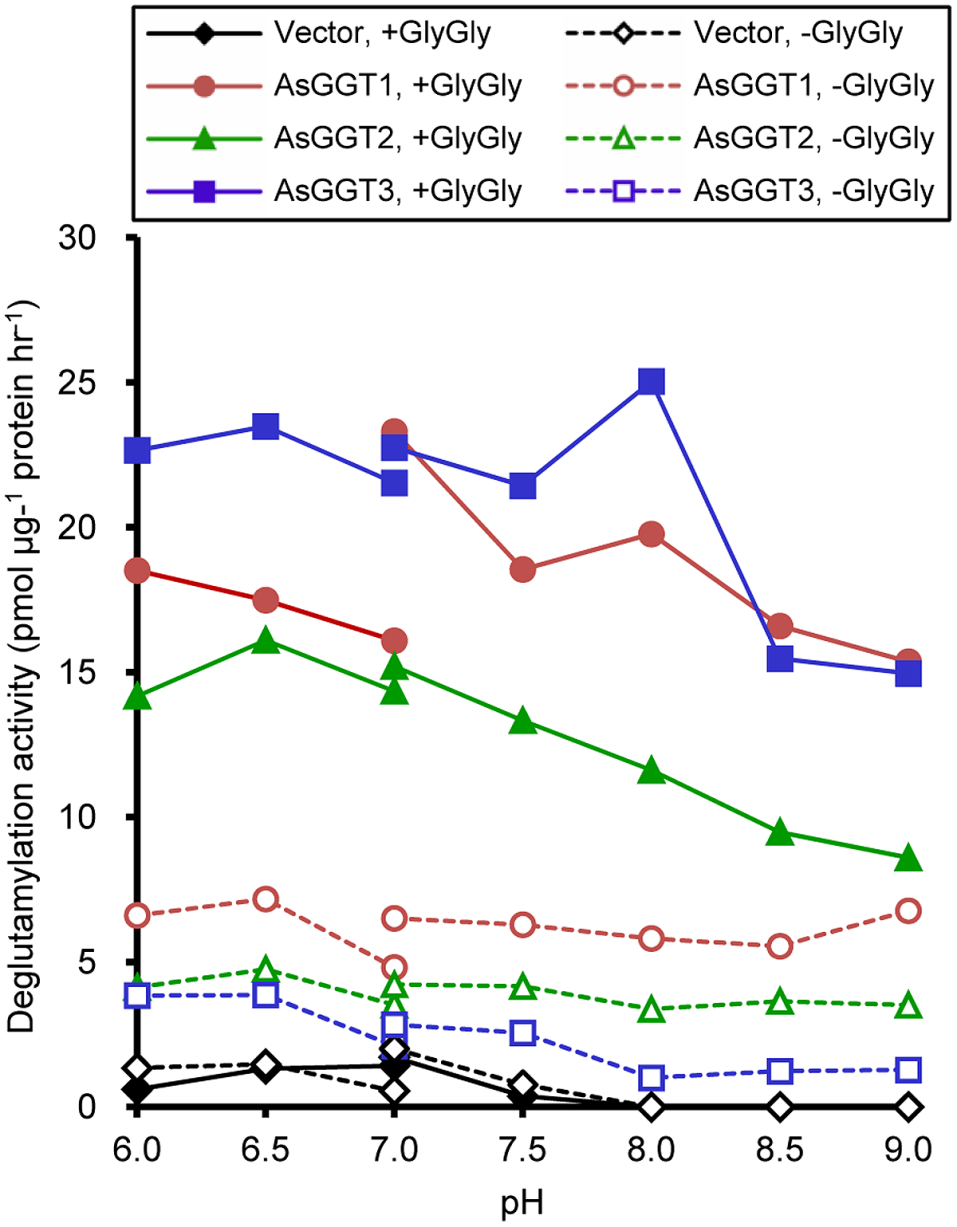
**pH dependence of transpeptidation and hydrolysis by AsGGT1, AsGGT2, and AsGGT3.** Production of *S*-allyl-L-cysteine from γ-glutamyl-*S*-allyl-L-cysteine was analyzed in the presence or absence of glycylglycine as the γ-glutamyl acceptor substrate. 2-(*N*-morpholino)ethanesulfonic acid buffer was used to cover the pH range from 6.0 to 7.0, and Tris-HCl buffer was used to cover the pH range from 7.0 to 9.0.

**FIGURE 6:**
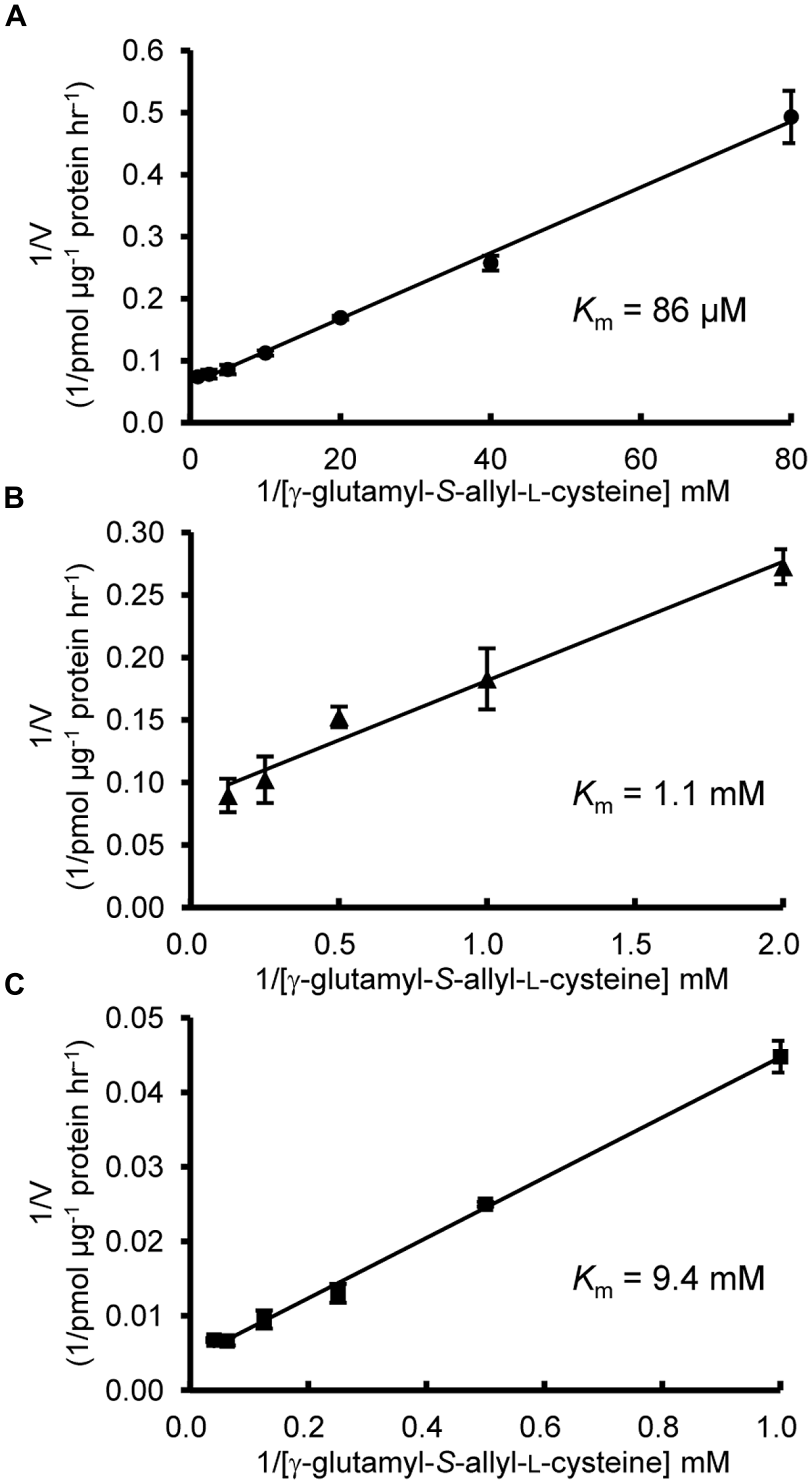
**Kinetic characterization of AsGGT1, AsGGT2, and AsGGT3**. Lineweaver-Burk plots between deglutamylation activity and γ-glutamyl-*S*-allyl-L-cysteine concentrations. Assays were carried out with γ-glutamyl-*S*-allyl-L-cysteine concentrations ranging from 12.5 to 1000 μM for AsGGT1 **(A)**, 0.5–8 mM for AsGGT2 **(B)**, and 1–25 mM for AsGGT3 **(C)**. Each data point represents the mean ± SD (*n* = 3).

Next, we examined the enzymatic activities of AsGGT1, AsGGT2, and AsGGT3 toward γ-glutamyl-*S*-allyl-L-cysteine and γ-glutamyl-*S*-allyl-L-cysteine sulfoxide, which are two possible biosynthetic intermediates in alliin biosynthesis, as potential γ-glutamyl donor substrates (**Figures 3** and **4**). Activities were measured in the presence of glycylglycine as a γ-glutamyl acceptor substrate. When γ-glutamyl-*S*-allyl-L-cysteine was used as a γ-glutamyl donor substrate, *S*-allyl-L-cysteine was not formed at a detectable level in assays using crude protein extracts from control yeast (**Figure 3**; **Table 1**), indicating that yeast endogenous GGT could not use γ-glutamyl-*S*-allyl-L-cysteine as a γ-glutamyl donor substrate. By contrast, considerable amounts of *S*-allyl-L-cysteine were detected in assays using crude protein extracts prepared from yeast cells expressing AsGGT1, AsGGT2, and AsGGT3 (**Figure 3**; **Table 1**), demonstrating that the recombinant proteins of AsGGT1, AsGGT2, and AsGGT3 can convert γ-glutamyl-*S*-allyl-L-cysteine to *S*-allyl-L-cysteine. The deglutamylation activities of AsGGT1, AsGGT2, and AsGGT3 toward γ-glutamyl-*S*-allyl-L-cysteine were decreased in the absence of glycylglycine (**Figure 5**), indicating that these garlic GGTs can catalyze transpeptidation more effectively than hydrolysis. In the presence of glycylglycine, the activities of AsGGT2 were higher with lower pH (**Figure 5**). Kinetic characterization of the recombinant AsGGT1, AsGGT2, and AsGGT3 exhibited typical Michaelis-Menten behavior, and the apparent *K*_m_ values of AsGGT1, AsGGT2, and AsGGT3 for γ-glutamyl-*S*-allyl-L-cysteine were 86 μM, 1.1 mM, and 9.4 mM, respectively, in the presence of glycylglycine (**Figure 6**).

When γ-glutamyl-*S*-allyl-L-cysteine sulfoxide was used as a γ-glutamyl donor substrate, only small amounts of alliin were detected in assays using crude protein extracts from yeast expressing AsGGT1 or AsGGT3 (**Figure 4**; **Table 1**), suggesting the recombinant AsGGT1 and AsGGT3 exhibit a weak activity to deglutamylate γ-glutamyl-*S*-allyl-L-cysteine sulfoxide. However, under the conditions we examined, the activities of AsGGT1 and AsGGT3 to deglutamylate γ-glutamyl-*S*-allyl-L-cysteine sulfoxide were too weak to perform further enzymatic characterization. AsGGT2 exhibited no detectable deglutamylation activity toward γ-glutamyl-*S*-allyl-L-cysteine sulfoxide (**Figure 4**; **Table 1**). These results indicate that AsGGT1, AsGGT2, and AsGGT3 are the functional GGT proteins with a preference for γ-glutamyl-*S*-allyl-L-cysteine as a γ-glutamyl donor substrate over γ-glutamyl-*S*-allyl-L-cysteine sulfoxide.

### SUBCELLULAR LOCALIZATION OF AsGGT1, AsGGT2, AND AsGGT3

The probable subcellular localization of AsGGT1, AsGGT2, and AsGGT3 was computationally analyzed using the program TargetP v1.1 ^3^ and WoLF PSORT ^4^. Both programs predicted that these three garlic GGT proteins lack signal peptides for secretion or localization to cellular organelles.

**FIGURE 7:**
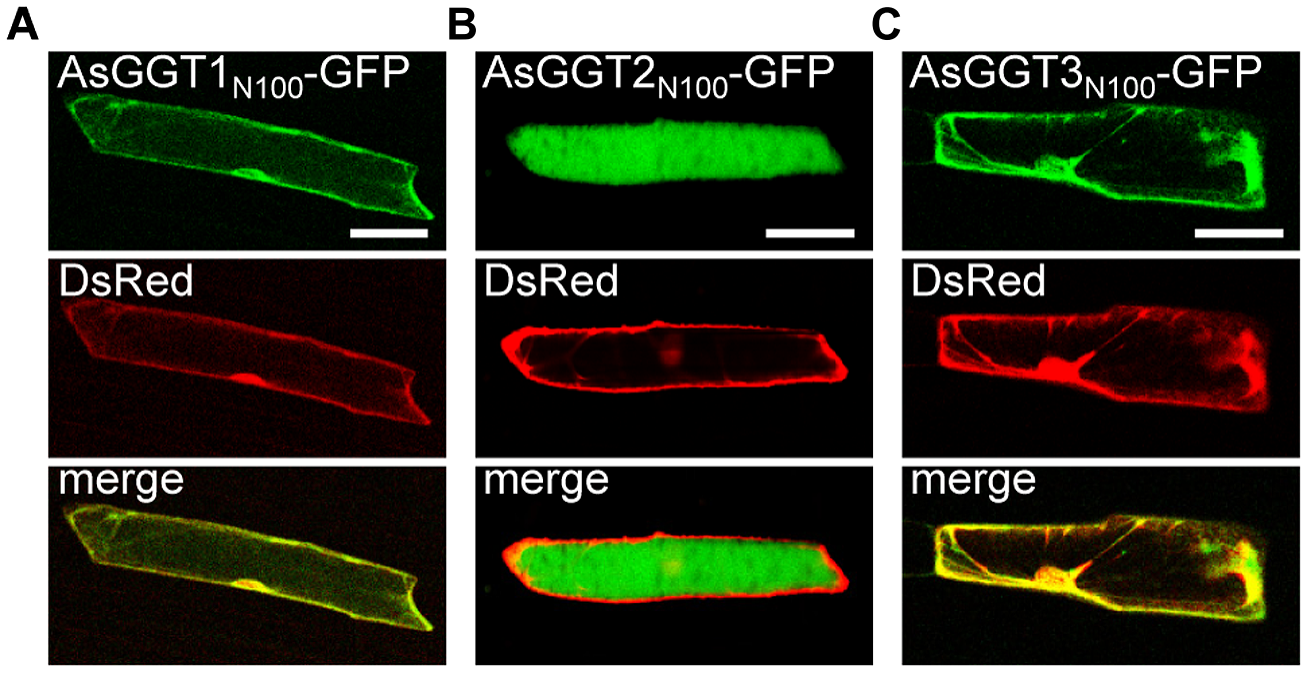
**Transient expression of GFP fusion proteins of AsGGT1, AsGGT2, and AsGGT3 in onion epidermal cells.** GFP fusion proteins, AsGGT1_N100_-GFP **(A)**, AsGGT2_N100_-GFP **(B)**, or AsGGT3_N100_-GFP **(C)**, were transiently co-expressed with DsRed in onion epidermal cells. DsRed was used as a control for cytosolic localization. Bars = 100 μm.

To determine subcellular localization of AsGGT1, AsGGT2, and AsGGT3, the green fluorescent protein (GFP)-fusion constructs of AsGGT1, AsGGT2, and AsGGT3 were transiently expressed in onion epidermal cells under the control of the cauliflower mosaic virus 35S RNA promoter by using the particle bombardment method. Since N-terminal sequences of AsGGT1, AsGGT2, and AsGGT3 are long and may contain the signal sequence for secretion or targeting to cellular organelles, three types of fusion proteins, GFP C-terminally fused to the N-terminal 100-amino acid residues of GGT (AsGGT1_N100_-GFP, AsGGT2_N100_-GFP, and AsGGT3_N100_-GFP), GFP C-terminally fused to the N-terminal 300-amino acid residues of GGT (AsGGT1_N300_-GFP, AsGGT2_N300_-GFP, and AsGGT3_N300_-GFP), and GFP C-terminally fused to the full-length GGT protein (AsGGT1_Full_-GFP, AsGGT2_Full_-GFP, and AsGGT3_Full_-GFP), were analyzed. As a control of cytosolic localization, DsRed protein was simultaneously expressed with each GFP-fusion protein. The green fluorescent signals derived from AsGGT1_N100_-GFP and AsGGT3_N100_-GFP overlapped with the red fluorescence of DsRed (**Figures 7A,C**), suggesting that N-terminal regions of AsGGT1 and AsGGT3 have no signal sequence for secretion or targeting to cellular organelles. Similarly, the green fluorescent signals from AsGGT1_N300_-GFP, AsGGT1_Full_-GFP, AsGGT3_N300_-GFP, and AsGGT3_Full_-GFP were observed in the cytosol, although the fluorescent signal intensities were much weaker (data not shown). By contrast, the green fluorescence from AsGGT2_N100_-GFP was observed predominantly in the vacuole (**Figure 7B**), indicating that AsGGT2 has a signal sequence for targeting to the vacuole within its N-terminal 100 amino acid residues. When AsGGT2_N300_-GFP and AsGGT2_Full_-GFP were expressed, weak fluorescent signals were detected both in the vacuole and cytosol (data not shown).

## DISCUSSION

In this study, we identified three novel genes encoding GGTs, *AsGGT1*, *AsGGT2*, and *AsGGT3*, from garlic by utilizing their partial sequence information found in a publicly available EST database or by utilizing sequence information of conserved regions of known plant GGTs. The deduced amino acid sequences of *AsGGT1*, *AsGGT2*, and *AsGGT3* contained threonine residues required for autoprocessing and the residues required for GGT activity (Ikeda et al., 1993, 1995a,b; Okada et al., 2006), suggesting that *AsGGT1*, *AsGGT2*, and *AsGGT3* all encode functional GGT proteins. The cDNA sequence of *AsGGT3* and the previously identified partial cDNA sequence of garlic *AsGGT* (Cho et al., 2012) were almost identical in their overlapping region, suggesting that these two cDNAs were derived from a single gene encoding GGT. The high sequence homology between garlic *AsGGT3* and onion *AcGGT* (Shaw et al., 2005) may indicate that they are orthologs. By contrast, *AsGGT1* and *AsGGT2* showed relatively low sequence similarity with garlic *AsGGT* (Cho et al., 2012) and onion *AcGGT* (Shaw et al., 2005). Phylogenetic analysis revealed that AsGGT3 belongs to a subgroup different from that containing AsGGT1 and AsGGT2 (**Figure 2**), suggesting that the biochemical characteristics of AsGGT3 might be somewhat different from those of AsGGT1 and AsGGT2.

The deglutamylation activities of AsGGT1, AsGGT2, and AsGGT3 toward alliin biosynthetic intermediates were demonstrated by *in vitro* biochemical assays, using recombinant proteins expressed in yeast. In the hypothetical alliin biosynthetic pathway, two different routes from the intermediate γ-glutamyl-*S*-allyl-L-cysteine to alliin are possible, according to differences in the order of deglutamylation and *S*-oxygenation reactions (**Figure 1**): a potential route via deglutamylation of γ-glutamyl-*S*-allyl-L-cysteine to yield *S*-allyl-L-cysteine that is further *S*-oxygenated to alliin, and an alternative route via *S*-oxygenation of γ-glutamyl-*S*-allyl-L-cysteine to form γ-glutamyl-*S*-allyl-L-cysteine sulfoxide that is further deglutamylated to yield alliin. Our results demonstrated that AsGGT1, AsGGT2, and AsGGT3 actively deglutamylate γ-glutamyl-*S*-allyl-L-cysteine, whereas these GGTs have almost no deglutamylation activity toward γ-glutamyl-*S*-allyl-L-cysteine sulfoxide (**Figure 4**; **Table 1**). It can be speculated that the intermediate γ-glutamyl-*S*-allyl-L-cysteine is mainly deglutamylated prior to being *S*-oxygenated in alliin biosynthesis in garlic. This hypothesis is also supported by our recent study on flavin-dependent *S*-oxygenase, which preferably utilizes *S*-allyl-L-cysteine, rather than γ-glutamyl-*S*-allyl-L-cysteine, as the substrate (unpublished results). The presence of dipeptide glycylglycine as a γ-glutamyl acceptor increased the deglutamylation activities of AsGGT1, AsGGT2, and AsGGT3 (**Figure 5**), showing that these GGTs catalyze transpeptiation more efficiently than hydrolysis, as in GGTs from *Arabidopsis* and onion (Storozhenko et al., 2002; Shaw et al., 2005).

There are two characteristic differences among the AsGGT1, AsGGT2, and AsGGT3 proteins. One is in their affinity for γ-glutamyl-*S*-allyl-L-cysteine (**Figure 6**). The apparent *K*_m_ values of AsGGT1 and AsGGT2 for γ-glutamyl-*S*-allyl-L-cysteine determined in this study (86 μM and 1.1 mM, respectively) are lower than or comparable to those of partially purified onion GGT for γ-glutamyl-*S*-propenyl-L-cysteine (*K*_m_ = 1.68 mM) and for γ-glutamyl-*S*-methyl-L-cysteine (*K*_m_ = 0.55 mM; Lancaster and Shaw, 1994). By contrast, AsGGT3 exhibited a relatively low affinity for γ-glutamyl-*S*-allyl-L-cysteine (*K*_m_ = 9.4 mM). This is in agreement with the results of a previous study that onion AcGGT, which shares high sequence homology with AsGGT3, could not utilize γ-glutamyl-*trans*-*S*-1-propenyl-L-cysteine sulfoxide (a major γ-glutamylated biosynthetic intermediate in onion) as a good γ-glutamyl donor substrate (Shaw et al., 2005). In garlic, the content of alliin is increased dramatically before and during the maturation of bulbs (Ueda et al., 1991). Alliin is largely found in leaves before the formation of bulbs and in the initial stage of bulb maturation, whereas it is found predominantly in bulbs in the later stage of bulb formation (Ueda et al., 1991; Koch and Lawson, 1996). It is suggested that leaves of garlic actively biosynthesize alliin before the formation of bulbs and in the initial stage of bulb maturation. However, in leaves at the same stages, γ-glutamyl-*S*-allyl-L-cysteine is present in trace levels (0.01 mg g^-1^ fresh weight; Matsuura et al., 1996). The concentration of γ-glutamyl-*S*-allyl-L-cysteine in cells of these tissues is calculated to be approximately 38 μM, when the content of water in tissues is estimated to be 90%. By contrast, the content of γ-glutamyl-*S*-allyl-L-cysteine in mature bulbs is ∼5 mg g^-1^ fresh weight (Matsuura et al., 1996; Ichikawa et al., 2006a,b), and the concentration of γ-glutamyl-*S*-allyl-L-cysteine in cells of bulbs is calculated to be 26 mM, when the content of water in tissues is estimated to be 65%. The highly accumulated γ-glutamyl-*S*-allyl-L-cysteine in bulbs is stored during dormancy of bulbs at -3°C, while it is rapidly converted to alliin when bulb dormancy is broken at 4°C (Ichikawa et al., 2006a). Based on these observations, we hypothesize that AsGGT1 and AsGGT2, which exhibit high-affinity for γ-glutamyl-*S*-allyl-L-cysteine, would contribute to the biosynthesis of alliin in leaves during the formation and maturation of bulbs, while AsGGT3 may contribute to alliin biosynthesis in bulbs during dormancy-breaking or, alternatively, the main *in vivo* function of AsGGT3 may not be the deglutamylation of γ-glutamyl-*S*-allyl-L-cysteine. The other major difference observed among AsGGT1, AsGGT2, and AsGGT3 is in their subcellular localization. Transient expression analyses of GFP-fused AsGGT2 proteins in onion cells suggested that AsGGT2 is predominantly localized in the vacuole *in vivo*. In contrast to the almost exclusive localization of AsGGT2_N100_-GFP in the vacuole (**Figure 7B**), the green fluorescence signals from AsGGT2_N300_-GFP and AsGGT2_Full_-GFP were detected both in the vacuole and cytosol (data not shown), suggesting that a part of AsGGT2_N300_-GFP and AsGGT2_Full_-GFP polypeptides was not properly processed and/or assembled and thus was not sorted to the vacuole in the heterologous expression system we used. Alternatively, AsGGT2 may localize both in the vacuole and cytosol in garlic cells. The signal sequence for targeting to the vacuole of AsGGT2 is located within its N-terminal 100 amino acids, as in *Arabidopsis* AtGGT4 (Grzam et al., 2007; Ohkama-Ohtsu et al., 2007b). To date, several sequence motifs for vacuolar targeting have been identified from plants (Xiang et al., 2013). However, we could not identify the potential motif for vacuolar targeting that is conserved between AsGGT2 and AtGGT4. Future studies are needed to determine the sequence motif and the mechanism for their targeting to the vacuole. Consistent with vacuolar localization of AsGGT2, the deglutamylation activities of AsGGT2 toward γ-glutamyl-*S*-allyl-L-cysteine were increased under weakly acidic conditions (**Figure 5**). AsGGT2 is suggested to contribute alliin biosynthesis mainly in the vacuole. In addition, AsGGT2 may function in the breakdown of glutathione *S*-conjugates in the vacuole in a similar manner as *Arabidopsis* AtGGT4 (Grzam et al., 2007; Ohkama-Ohtsu et al., 2007b). In contrast, GFP-fusion proteins of AsGGT1 and AsGGT3 were retained in the cytosol (**Figures 7A,C**), suggesting that AsGGT1 and AsGGT3 have no apparent signal sequence for targeting to the cellular organelles in their N-terminal peptides. To the best of our knowledge, there have been no reports of GGT proteins localizing in the cytosol. Future investigations will reveal whether AsGGT1 and AsGGT3 are new types of GGT proteins that localize and function in the cytosol *in vivo* or not. To date, the subcellular distribution of alliin biosynthetic intermediates and enzymes in garlic remains largely unclear, although the previous cell fractionation experiment suggested that γ-glutamyl peptides and *S*-alk(en)yl-L-cysteine sulfoxides are mainly located in the cytosol in onion (Lancaster et al., 1989). Our results suggest that AsGGT1, AsGGT2, and AsGGT3 contribute differently to alliin biosynthesis, according to differences in their kinetic properties and localization patterns. Recently, five γ-glutamyl peptidases (GGPs), which have similar catalytic functions but no sequence homology with GGTs, were identified from *Arabidopsis*. Among these, GGP1 and GGP3 were shown to be cytosolic proteins that play major roles in the removal of γ-glutamyl groups from glutathione *S*-conjugates in the biosynthesis of glucosinolates and camalexins (Geu-Flores et al., 2009, 2011). Although identification of GGPs from garlic has not been reported to date, it is likely that GGPs exist and function in the deglutamylation reaction in alliin biosynthesis, perhaps together with GGTs.

In the present study, we succeeded in identifying three garlic GGTs, AsGGT1, AsGGT2, and AsGGT3, that can deglutamylate an alliin biosynthetic intermediate, γ-glutamyl-*S*-allyl-L-cysteine. Future investigations of the *in vivo* functions of AsGGT1, AsGGT2, and AsGGT3 will provide a better understanding of the molecular mechanisms underlying the biosynthesis of alliin in garlic, which can be applied to future metabolic engineering of plants.

## Conflict of Interest Statement

The authors declare that the research was conducted in the absence of any commercial or financial relationships that could be construed as a potential conflict of interest.

## Footnotes

1 http://garlicdb.kribb.re.kr

2 http://garlicdb.kribb.re.kr

3 http://www.cbs.dtu.dk/services/TargetP/

4 http://wolfpsort.org/
